# Comparative Assessment of Different Health Utility Measures in Systemic Lupus Erythematosus

**DOI:** 10.1038/srep13297

**Published:** 2015-08-21

**Authors:** Su-li Wang, Evelyn Hsieh, Li-an Zhu, Bin Wu, Liang-jing Lu

**Affiliations:** 1 Department of Rheumatology, Ren Ji Hospital, School of Medicine, Shanghai Jiao Tong University, Shanghai, China; 2 Yale School of Medicine, The Anlyan Center, New Haven, Connecticut, United States of America

## Abstract

In a time of increasing economic constraints, it is crucial that health systems optimize their resource use to ensure that they generate the maximum possible health gain. Therefore, it is necessary for health interventions to be evaluated and compared across therapeutic boundaries. Undertaking such an evaluation a generic utility-based measure is required. But it remains uncertain whether the utility values obtained by direct or indirect methods are comparable and which approach is the most appropriate in Systemic Lupus Erythematosus (SLE) population. In the study, we compared the utility values obtained by an indirect method (EQ-5D) with direct utility instruments, the standard gamble (SG) and visual analog scale (VAS), in SLE patients. The correlations between VAS, EQ-5D and LupusQoL were significant; relative good intraclass correlations or kappa coefficients indicated the reliability of these instruments. A model incorporating the SLEDAI scores and LupusQoL domains of emotional health and pain was a good predictor of VAS. SLEDAI score was a good predictor in the SG regression model. These findings suggested that the VAS and EQ-5D might be valid and reliable measures to assess health related quality of life in SLE patients and represent promising outcome measures for future research in this population.

Systemic lupus erythematosus (SLE) is a heterogeneous autoimmune disease, which has posed a significant challenge to rheumatologists in terms of diagnosis and treatment. Rapid advances in medical knowledge and treatment regarding rheumatic diseases have increased survival in patients with SLE, leading to a shift in attention towards the long-term burden that this disease imposes upon patients, healthcare systems and society^1^,^2^,^3^. Prior studies have shown that health-related quality of life (HRQoL) among patients with SLE is worse than the general population, particularly those diagnosed at an earlier age, and that this poorer HRQoL persists over long-term follow-up^3^,^4^,^5^. As SLE an incurable chronic condition that predominantly affects women of working age, it brings not only a heavy health burden to patients themselves, but also heavy economic burdens to their families, societies and governments^6^,^7^. In a time of increasing economic constraints, it is crucial that health systems optimize their resource utilization to ensure that health gains are maximized. Therefore, it is necessary for health interventions to be evaluated and compared across therapeutic boundaries. The need for more sophisticated economic analyses will continue to grow, especially in light of the number of novel therapies that have entered the market or that are currently under investigation for SLE. Quality adjusted life years (QALYs), fundamental concept in health economics, attempts to value a given health condition or situation in terms of a measure that reflects both the impact on longevity and quality of life in relation to the common numeraire of a year in full health^8^. Furthermore, the disease-specific utility score is the basis for the calculation of QALYs and the premise for valuing the benefits of certain forms of health care.

In the context of HRQoL measurement, utility is used to refer to the preference of the rater (usually a patient or a member of the general public) for a particular health outcome attribute^8^. Preference-based approaches integrate different aspects of health into utility score, a single index anchored by a value of 1 for optimal health and 0 for death (health states considered worse than death can be represented by negative values)^9^. Utility preference scores are key elements in cost-effectiveness research, which can be conducted in a number of ways, including using various “off the shelf” instruments [Health Utilities Index Mark 3 (HUI-3, http://www.healthutilities.com; Health Utilities Inc, Hamilton, Ontario, Canada), EuroQol (EQ-5D, http://www.euroqol.org; EuroQol Group, Rotterdam, the Netherlands), and Short Form 6D (SF-6D, http://www.qualitymetric.com; Quality Metric Inc, Lincoln, RI)] or direct instruments [visual analog scale (VAS), standard gamble (SG) and time trade-off(TTO)]^10^. To date there is no consensus on which method is the most appropriate^11^, however, if the scores produced by different methods differ significantly, this can impact estimates of cost-effectiveness obtained and may lead to discrepant or uncertain conclusions as to whether or not an intervention should be recommended or funded.

In present study, we address the issue of concurrent validity and reliability of the following utility or preference measures in patients with SLE: VAS, SG, and EQ-5D. We also examine the ability of these measures to distinguish between subgroups of individuals with different levels of SLE severity, and compare them to a disease-specific instrument, LupusQoL. The predictors of utility of VAS and SG were determined by multiple regression models.

## Methods

This study was approved by the Institutional Review Board of Shanghai Jiao Tong University and the Ethics Committee of Ren Ji Hospital, and all subjects consented to participation. The methods were carried out in accordance with the approved guidelines. All research was performed on the basis of the principles expressed in the Declaration of Helsinki.

### Patients

245 consecutive SLE patients, who were followed at the Ren Ji Hospital, School of Medicine, Shanghai Jiao Tong University from March 2013 to May 2014 were included. All enrolled patients satisfied the 1997 revised American College of Rheumatology classification criteria for SLE^12^, and were on a stable treatment regimen for at least 2 months at the time of entry into the study. Each patient took part in at least one standardized interview with the same trained interviewer. 100 patients were selected randomly to participate in a second interview within a 2 to 4 week interval. At each interview, all patients underwent assessments of disease activity [SLE Disease Activity Index (SLEDAI)] and damage [Systemic Lupus Collaborating Clinics/American College of Rheumatology Damage Index (SLICC/ACR DI)], and completed a disease-specific HRQoL measure, LupusQoL, and 3 utility measures: VAS, SG, and EQ-5D. The questionnaires were self-administered on the same day as the baseline evaluation. Questionnaires were administered in a different order each day to minimize the effect of sequence on the outcomes of interest.

### Utility measures

#### EQ-5D

EQ-5D is a generic preference-based measure of health developed by a multidisciplinary group of researchers, and is one of the most popular methods for obtaining health state values to calculate QALYs^13^,^14^. This instrument has a structured health state descriptive system with five dimensions (mobility, self-care, usual activities, pain/discomfort and anxiety/depression) and the EQ visual analogue scale (EQ VAS). There are two versions of the questionnaire, one with five-level responses and one with three-level responses. We chose to use the version with five levels, EQ-5D-5L (no problems, slight problems, moderate problems, severe problems, and extreme problems), because it has improvement in discriminatory power and is considered more user-friendly. These five dimensions together define a total of 5^5^ health states formed by different combinations of levels. EQ-5D-5L health states, defined by the EQ-5D-5L descriptive system, may be converted into utility values according to country specific value sets. Previous research has already demonstrated the construct and criterion validity in SLE patients^15^.

#### VAS

The VAS is usually represented by a line with well-defined end-points, on which respondents can indicate their judgments, values or feelings. It has been used in context of health as a measure of symptoms and various domains of health, and to provide a single index measure of HRQoL. This measure has been identified as a possible economic evaluation tool for over four decades, and has become one of the most widely used measures for economic purposes^16^. In this study, we obtained the VAS utility by using EQ-5D-5L VAS. Using interval markings of 0–100, where 0 corresponded to the worst imaginable health and 100 corresponded to the best imaginable health, patients were asked to indicate their state of health on the day of interviews. Reported VAS scores were divided by 100 prior to analysis to make the scores comparable to the 0–1 scale of the utility score.

#### SG

The SG, one of best known approaches to explicit assessment of preferences, is regarded as the gold standard for eliciting utilities due to its grounding in utility theory. Patients in our study were asked repeatedly to choose between the certain intermediate outcome and the gamble with varied probability (perfect health or death) until they were indifferent between the two alternatives^8^. This probability was the utility for this health state.

### Disease specific HRQoL questionnaire

LupusQoL is a lupus-specific HRQoL questionnaire consisting of 34 items grouped in eight domains: physical health (PH), pain (PN), planning (PL), intimate relationships (IR), burden to others (BU), emotional health (EH), body image (BI) and fatigue (F)^17^. This tool was modified and validated for applicability to Chinese patients with SLE in our previous study^18^.

### Statistical analyses

Spearman’s correlations were calculated between the LupusQoL domains and the utility measures of the first visit to assess validity.

Patients were divided into two groups by a SLEDAI score cutoff of 4 and SLICC-DI score cutoff of 1 respectively. To test the construct validity of these utility measures, we compared the utility scores between these two groups by using the Kruskal-Wallis test. Our hypothesis was that utility scores of patients would be altered in these two groups.

To assess reliability, intraclass correlations for VAS and SG or kappa coefficients for EQ-5D were calculated between first and second assessments, performed 2–4 week apart, in patients whose self-assessed quality of life was rated as no change on a 15-point health status change scale (−7 to +7).

Multiple regression models were performed for VAS and SG to determine predictors of utility. Variables included in the model were SLEDAI, SDI, and selected domains of LupusQoL which were considered to best characterize the values of patients’ health states. We selected the best models according to Approximate Bayes Factors calculated by the Bayesian Information Criterion (BIC), which have better out of sample predictive properties compared to backwards or forwards model selection algorithms.

Categorical data were compared between groups using Pearson and Fisher’s exact tests as appropriate and continuous variables were compared using analysis of variance or Kruskal-Wallis, as appropriate. Correlations were evaluated with Spearman’s or Pearson’s correlation tests, as appropriate. A strong correlation was defined as ≥0.70, moderate to substantial as 0.30–0.70 and weak as <0.30. All reported p values were 2-sided and p values <0.05 were considered statistically significant. We used SPSS software, version 10.0 to analyze data. Multiple regression models analysis was programmed in R software environment (version 3.1.1; R Development Core Team, Vienna, Austria).

## Results

### Demographics and baseline characteristics

245 respondents with confirmed SLE completed the baseline assessments. Complete data were available from 220. 93.6% (n = 206) were women; all were Chinese. The mean (SD) age and disease duration were 33.70 (9.20) years and 4.76 (4.40) years. The mean (SD) SLEDAI and SDI were 3.02 (4.04) (median 2, range 0–25) and 0.34 (0.87) (median 0, range 0–6), respectively (Table 1).

The mean (SD) of the three health utility measures are shown in Table 2. The strength of the correlations between the utility scores of the three measures and the eight LupusQoL domains have been shown in Table 3. We found significant positive correlations between EQ-5D score and all domains of LupusQoL (p values < 0.01). The correlations were moderate to strong (0.4–0.7) for all domains except intimate relationship (r = 0.252) and body image (r = 0.179). A similar situation could be found in the correlation between VAS scores and domains of LupusQoL. However, SG scores correlated poorly with six domains of LupusQoL (maximum r = 0.293; minimum r  = 0.151). The remaining two domains, burden to others and body image did not correlate significantly with SG scores (r = 0.104 and 0.057).

All three utility measures demonstrated the ability to discriminate patients with lower versus higher disease activity (SLEDAI 0–4 versus >4) and damage scores (SDI ≤1 versus > 1). The utilities differences of these two groups were significant (Table 4).

The intraclass coefficient by comparing scores at baseline and 2 or 4 weeks in 100 patients who rated their own quality of life as no change were good for the VAS [ICC = 0.793, 95% CI (0.707, 0.856)] and SG [ICC = 0.770, 95% CI (0.676, 0.839)]. The kappa coefficient was poor (0.284) for the EQ-5D domain of Anxiety/Depression, but excellent for the remaining domains (Mobility: 0.786; Self-care: 0.849; Usual activities: 0.972; Pain/Discomfort: 0.796).

Analysis of multiple regression models showed that a model that integrated the SLEDAI scores, pain and emotional health domains of LupusQoL was a good predictor of VAS (R^2^ = 0.543) (Table 5). SLEDAI score was also a good predictor in the regression model for SG (R^2^ = 0.226) (Table 5).

## Discussion

Utility scores are widely used to compare the effect of different disease states on overall functional status and quality of life. They are also essential tools for comparing the cost-effectiveness of different treatments for a given disease. Both direct and indirect measures can be used to obtain utility values. They both have a solid theoretical basis, each having its advantages and disadvantages^8^. However, it remains uncertain whether the utility values obtained these methods are comparable and which approach is the most appropriate for patients with SLE. This is the first study to examine the construct validity of three such instruments simultaneously in a relatively large sample of participants with SLE.

Sufficient data in our study are available indicating that three utility measures can be used for assessment of health status in patients with SLE. The mean utility values derived from three methods, VAS, SG and EQ-5D, were at a comparable level.

However, similarly to the previous studies, each data of these methods displayed relative large standard deviation (SD). The most possible reason is that rheumatic diseases, especially SLE, have extremely variable health states. SLE, as a chronic disease with easy recurrence, has a wide spectrum of clinical manifestation. These disease characteristics were well reflected in the utility values.

In our study, the VAS and EQ-5D measures show better overall correlation with LupusQoL compared with SG instrument. The VAS and EQ-5D all reflected the general health status of SLE patients, and their correlation with the domains reflecting general health status (e.g. physical health, pain, planning and fatigue) were stronger. By contrast, SG was not significantly correlated with the disease-specific domains for SLE (burden to others and body image), and the correlations between SG and the remaining domains of LupusQoL were also weak.

Based on the axioms of expected utility theory, SG has traditionally been viewed as the “gold standard” for the measurement of the utility associated with particular health states^8^. According to the basic principles of SG, participants in a hypothetical are presented with the risk of immediate death when making a choice between two offered alternatives, which generally leads patients to choose the more conservative strategy. As a result, the findings deduced with SG may theoretically be slightly higher than other methods^19^. However, in our study the mean utility of SG was founded to be lower than VAS and EQ-5D. This finding casts some doubt regarding applicability of SG in this patient population. In China, patients living with chronic illnesses commonly face formidable challenges securing employment, attaining education and marriage, and suffer from discrimination in society. As such, some patients may have a strong desire to rid themselves of chronic disease, even at the cost of valuable life. We believe this kind of psychology may have had a significant impact on our results. Furthermore, although it is well established that the SG is normatively more validity than the other methods of health utility measurement, but its descriptive validity is less certain^8^. Some experts therefore recommend utilities derived from SG should be adjusted for probability weighting using a weighting function, thereby improving the descriptive validity without sacrificing normative validity^20^.

As indicated by good intraclass correlations between the two interviews, these three methods, VAS, SG and EQ-5D are all reliable in our population. Only one domain, anxiety/depression in EQ-5D, was found to be less reliable. However, the emotional states of patients with SLE such as anxiety and depression can be affected by many factors, some of which are associated with disease states and others are not. Therefore, it is possible that some patients who were in relatively stable disease states could experience emotional changes over a 2 to 4 weeks period due to other reasons.

A model integrated the SLEDAI scores, pain domain and emotional health domain of LupusQoL was a good predictor of VAS, suggesting that the health utility score depends not only on physical levels of lupus activity, but also on the patients’ state of mental health as well. The finding also demonstrated that pain is one of the most important factors impacting quality of life in this population.

This study had a few important Limitations. All patients were recruited from a single clinical site in China, which limits the generalizability of our findings. However, as one of the most reputable departments of rheumatology in China, our patients population consisted of diverse range of SLE cases referred from a large catchment area. It should mitigate the influence of the weakness to our results. We also did not take the measurement of the minimal clinically important difference for each instrument. Further studies to measure the responsiveness of utility measurement to change following treatment of SLE are also required.

## Conclusions

The results of this study lend support to the construct validity of two instruments (VAS, EQ-5D) in SLE and provide some detail regarding their limitations and strengths. SG, if it is adjusted for probability weighting, might become a more efficient method for utility calculation in SLE however this requires further examination. Our study provides a foundation for future studies evaluating utility data with larger samples of patients with SLE and provides an important theoretical approach for the reasonable allocation of health resources for this population.

## Additional Information

**How to cite this article**: Wang, S.- *et al.* Comparative Assessment of Different Health Utility Measures in Systemic Lupus Erythematosus. *Sci. Rep.* **5**, 13297; doi: 10.1038/srep13297 (2015).

## Figures and Tables

**Table 1:** Patients’ baseline characters.

	All participants	Double-participants	P
N	220	100	
Age, years mean(SD)	33.70(9.20)	33.28(8.35)	0.53*
Female, N (%)	206(93.6)	89(89)	0.06#
Disease duration, years mean(SD)	4.76(4.40)	5.28(4.89)	0.18*
SLEDAI, median (range)	2(0, 25)	1(0, 11)	0.001#
SDI, median (range)	0(0, 6)	0(0, 2)	0.002#

SDI, SLICC/ACR damage index; SLEDAI, SLE Disease Activity Index.

^*^T-test.

^#^non-parameters tests.

**Table 2:** Utility values of 3 measures.

Utility Values	Mean(SD)	Median (range)
VAS	0.743(0.171)	0.800(0.10, 1.0)
SG	0.669(0.251)	0.750(0.0, 0.950)
EQ-5D	0.805(0.186)	0.814(−0.086, 1.0)

VAS: visual analog scale; SG: standard gamble; EQ-5D (EuroQol scale); SD: standard deviation.

**Table 3:** Spearman’s correlation between LupusQoL domains and utilities.

LupusQoL Domains	VAS	SG	EQ-5D scores
Physical health	0.417**	0.293**	0.603**
Pain	0.436**	0.246**	0.703**
Planning	0.416**	0.241**	0.446**
Intimate relationship	0.265**	0.163*	0.252**
Burden to others	0.314**	0.104	0.437**
Emotional health	0.380**	0.151*	0.576**
Body image	0.212**	0.057	0.179**
Fatigue	0.316**	0.204**	0.544**

VAS: visual analog scale; SG: standard gamble; EQ-5D (EuroQol scale); **p < 0.01, *p < 0.05.

**Table 4:** Disciriminant validity of utility measurements with SLEDAI and SDI as the external anchor.

Utility	Disease activity according to SLEDAI	Disease damage according to SDI				
	0–4	>4	P value	≤1	>1	P value
VAS	0.786	0.561	0.00	0.780	0.597	0.00
SG	0.727	0.442	0.00	0.708	0.534	0.01
EQ-5D	0.846	0.612	0.00	0.837	0.663	0.00

VAS: visual analog scale; SG: standard gamble; EQ-5D (EuroQol scale); SDI, SLICC/ACR damage index; SLEDAI, SLE Disease Activity Index.

**Table 5:** Regression models for predictors of utility.

Utility	Predictors	R^2^ Value	Parameter Estimate
VAS	SLEDAI	0.543	−0.0126
	Pain Domain		0.0244
	Emotional health Domain		0.0153
SG	SLEDAI	0.2262	−0.0178

VAS: visual analog scale; SG: standard gamble; SLEDAI, SLE Disease Activity Index.
